# Dental and Periodontal Treatment Need after Dental Clearance Is Not Associated with the Outcome of Induction Therapy in Patients with Acute Leukemia: Results of a Retrospective Pilot Study

**DOI:** 10.1155/2020/6710906

**Published:** 2020-04-21

**Authors:** Gerhard Schmalz, Lulzim Tulani, Rilana Busjan, Rainer Haak, Tanja Kottmann, Lorenz Trümper, Justin Hasenkamp, Dirk Ziebolz

**Affiliations:** ^1^Dept. of Cariology, Endodontology and Periodontology, University of Leipzig, Leipzig, Germany; ^2^Clinical Research Organization, Hamm, Germany; ^3^Clinic for Hematology and Medical Oncology, University Medical Center Goettingen, Göttingen, Germany

## Abstract

This retrospective pilot study aimed to detect whether remaining dental/periodontal treatment need and periodontal inflammation after dental clearance would be associated with the initial therapy outcome of adult patients with acute leukemia undergoing induction chemotherapy. Different parameters were assessed from the patients' records: initial blood parameters, blood parameters during initial chemotherapy, leukemia/therapy related complaints, duration of fever, microbiological findings (blood and urine), as well as patients' survival. Dental treatment need was defined as the presence of at least one carious tooth; periodontal treatment need was determined by the presence of probing depth ≥3.5 mm in at least two sextants. To reflect periodontal inflammation, the periodontal inflamed surface area (PISA) was applied. Thirty-nine patients were included. A dental treatment need of 75% and periodontal treatment need of 76% as well as an average PISA of 153.18 ± 158.09 were found. Only two associations were detected: periodontal treatment need was associated with thrombocyte count after 7 days (*p*=0.03), and PISA was associated with erythrocyte count three days after induction of therapy (*p*=0.01). It can be concluded that remaining dental and periodontal treatment need as well as periodontal inflammation after dental clearance is not associated with the outcome of induction therapy in adult patients with acute leukemia.

## 1. Introduction

Acute leukemia is a highly progressive and severe disease which takes a fatal course if no appropriate treatment is applied [1]. Thereby, induction chemotherapy is still the standard therapy for the majority of affected patients [2]. Although this therapeutic intervention is often the only chance to save patients' life, infectious complications caused by the immunosuppressive effect of chemotherapy are a major problem, leading to increased mortality [3]. As one potential entry point of bacteria in the blood, the oral cavity and related infectious diseases are of relevance as an important source of bacteraemia [4]. In consequence, the dental clearance prior to chemotherapy is a regular recommendation [5, 6]. This is supported by the fact that especially invasive oral treatment measures of patients undergoing chemotherapy can cause serious consequences [7]. Several considerations must be made in this context. The underlying leukemic disease can often lead to the affection of haematopoiesis, resulting in pancytopenia (anaemia, granulocytopenia, and thrombocytopenia) and thus to increased susceptibility to infections and increased bleeding tendency [1, 8, 9]. Furthermore, a delay in anticancer therapy caused by dental rehabilitation should be avoided [6]. These factors are important limitations of possible dental surgical interventions before the onset of induction chemotherapy. Thus, dental clearance is often primarily restricted to the elimination of dental foci with an increased risk of causing systemic complications [5, 10].

An available decision analysis model showed that additional death of 0.18% of patients can be avoided by dental treatment prior to chemotherapy, and a reduction in systemic infections by approximately one-third might be achievable [5, 10]. However, the clinical data supporting these findings are rare, especially regarding the effect of remaining treatment need after dental clearance. Recent examinations showed that, on the one hand, oral diseases might show a limited increase in the risk of bacteraemia in these patients [11]. On the other hand, it has been found that poor oral health had no influence on the outcome of induction therapy [12]. Accordingly, the real clinical impact of oral health situation on infectious complications and therapy outcome seems not clarified. In this context, increased risk during dental procedures in patients before chemotherapy [6] and a potential reduction of patients' quality of life by radical dental rehabilitation (e.g., numerous tooth extractions) [13] must be considered in the dental care of these patients.

The current pilot study was performed to detect whether a remaining treatment need (dental and periodontal) as well as periodontal inflamed surface area (PISA) after a previously performed dental clearance would affect the outcome of induction chemotherapy in adult patients with acute leukemia. It was hypothesized that remaining treatment need and periodontal inflammation after dental clearance would not affect therapy outcome and mortality.

## 2. Materials and Methods

### 2.1. Study design

The current examination was performed as a retrospective, observational pilot study and has been reviewed and approved by the Ethics Committee of the University Medical Center Goettingen, Germany (No. 30/1/14). All Patients provided their written informed consent for participation in the current study. All participants were already part of a cross-sectional evaluation of oral health and microbiological parameters in adult patients suffering from newly diagnosed acute leukemia. Accordingly, general oral health parameters and microbiological findings have already been published before [14].

### 2.2. Patients

Between January 2015 and April 2015, hospitalized patients suffering from acute leukemia in the Clinic of Hematology and Oncology of the University Medical Center Goettingen to receive induction chemotherapy were recruited. The assessment of clinical oral parameters was performed by one experienced dentist in course of the previous cross-sectional study [14]. Out of these patients, the participants for this retrospective analysis were selected using specific inclusion and exclusion criteria. Accordingly, a mandatory condition for participation in the current study was a history of induction chemotherapy because of acute leukemia in the Clinic of Hematology and Oncology of the University Medical Center Goettingen. Before the onset of chemotherapy, a dental clearance of potential infectious dental foci was performed by the dentist of included patients. Thereby, dental conditions that pose a risk for immediate acutization or systemic spreading should be eliminated prior to onset of induction therapy. The oral examination for this current study was performed after the patient´s appointment for dental clearance. The exclusion criteria were equal as in the previous cross-sectional examination: age below 18 years, a poor general health condition that prohibited oral examination, pregnancy, and autoimmune diseases such as rheumatoid arthritis or chronic bowel diseases.

### 2.3. Acquisition of medical and leukemia related data

The patients' records of all participants were screened, and the following parameters were extracted for analysis:

- Initial blood parameters before therapy onset: C-reactive protein (CRP), lactate dehydrogenase (LDH), activated partial thromboplastin time (aPTT), hemoglobin, hematocrit, and count of erythrocytes, leukocytes, and thrombocytes
- Course of blood parameters during initial therapy: count of erythrocytes, leukocytes, and thrombocytes at three and seven days after therapy onset
- Duration of thrombopenia, erythropenia, leukopenia, neutropenia, and fever during initial therapy
- The highest CRP value during initial therapy period (CRP max)
- The detection of positive microbiological findings in the culture of blood and urine
- leukemia/therapy related complaints including weakness, tachycardia, vertigo/nausea, and dyspnea
- death of participants during therapy and the reason why participants died

### 2.4. Oral Examination

The course of oral examination has been performed once under standardized conditions, as described previously [14]. In brief, dental health assessment included decayed (D), missing (M), and filled (F) teeth index (DMF-T) according to WHO [15]. Based on the presence of carious teeth showing cavitation of the tooth surface, which required dental intervention, dental treatment need was defined. Furthermore, the periodontal status included the periodontal probing depths (PPD), the occurrence of bleeding (Bleeding on probing = BOP), as well as the clinical attachment loss (CAL). According to the periodontal screening index (PSI), the presence of PPD ≥3.5 mm in at least two sextants was interpreted as a periodontal treatment need [16]. Furthermore, the periodontal inflamed surface area (PISA), which enables quantification of inflammatory periodontal burden, was assessed [17].

### 2.5. Statistical Analysis

Statistical analysis was performed using SPSS for Windows, version 24.0 (SPSS Inc., USA). The samples were tested for normal distribution with the Shapiro–Wilk test. In a comparison of two independent, normally distributed samples, a *t*-test was used, while nonnormally distributed samples were analyzed with Mann–Whitney *U*-test. Categorized data were analyzed using Fisher´s exact tests or chi-square tests, respectively. The significance level was set at *α* = 5%.

## 3. Results and Discussion

### 3.1. Patients, Treatment Need, and PISA

A total of 39 patients suffering from acute leukemia with a mean age of 55.61 ± 17.01 years were included in the current study. A dental treatment need was found for 75% of study participants, while 76% had a need for periodontal treatment. An average PISA of 153.18 ± 158.09 was determined for the patients (Table 1).

### 3.2. Associations between Treatment Need/PISA and Leukemia Parameters

For dental treatment need, no associations to the initial blood values and the initial therapy outcome parameters were found. Furthermore, the positive microbiological culture in blood and urine was not related to the presence of dental treatment need (*p* > 0.05; Table 2). Patients with periodontal treatment need showed a statistically significant lower thrombocyte count at seven days after therapy onset (43.0 ± 44.5 vs. 131.3 ± 108.5, *p*=0.03). Further associations between periodontal treatment need and blood parameters, therapy related factors, and microbiological findings could not be detected (*p* > 0.05; Table 2). Based on the distribution by the median of PISA, participants with a PISA >98 mm^2^ had a lower erythrocyte count at three days after induction of therapy (3.0 ± 0.5 vs. 3.9 ± 1.5, *p*=0.01). Further associations for PISA with blood values or therapy related parameters were not found (*p* > 0.05; Table 3).

### 3.3. Associations between Treatment Need/PISA and Complaints

For the assessed treatment related complaints, including weakness, tachycardia, vertigo/nausea, and dyspnea, no associations to either dental/periodontal treatment need or to the PISA were detected (*p* > 0.05; Table 4).

### 3.4. Associations between Treatment Need/PISA and Mortality

From the 39 included patients receiving induction chemotherapy, 6 patients died within the observational period. Half of them (*n* = 3) died because of the cancerous disease itself, while the cause of death for two other patients was an infection. The presence of dental or periodontal treatment need as well as the PISA was not associated with the mortality of participants (*p* > 0.05; Table 4).

## 4. Discussion

### 4.1. Summary of the Main Results

Despite a previous dental clearance, dental and periodontal treatment need of the examined patients with acute leukemia was high. No substantial associations of dental/periodontal treatment need as well as PISA with initial blood parameters, outcome parameters of initial therapy, complaints of the patients, microbiological findings, and mortality were found.

### 4.2. Comparison with Available Literature

Due to severe immunosuppression because of the underlying disease as well as its therapy, patients with acute leukemia are at increased risk for severe infections [3]. In this respect, bacteraemia from different sources are an important concern. Oral diseases, especially periodontal inflammation, are associated with both growth of bacteria with high pathological potential and increased permeability of inflamed epithelial surface [18]. These factors enable a large amount of bacteria to enter the blood circulation, even during daily routine procedures like chewing or oral hygiene measures [19, 20]. High prevalence of gingivitis/periodontitis was concluded to be an independent risk factor for infectious complications during chemotherapy [21]. Furthermore, dental interventions, especially oral surgery during chemotherapy, can cause serious consequences like bleeding complications [7]. Accordingly, dental rehabilitation prior to induction chemotherapy is recommended to avoid both infectious complications and the necessity of dental interventions during therapy [5, 6]. This is also supported by a survey of dental clinicians, which prioritize dental rehabilitation before induction therapy [22].

The previous cross-sectional study of this working group found a poor oral health condition in adult patients with untreated acute leukemia and concluded improved dental care to be necessary for these patients [14]. However, it was unclear whether this oral situation would affect the therapy outcome, occurrence of infections, or mortality of the patients. Therefore, the current study retrospectively assessed these parameters. As the first aspect, there were no associations for treatment need (dental and periodontal) and PISA with initial hematological blood parameters. These findings are in line with an examination by Angst et al. [23]. In generally healthy individuals, the reflection of periodontal inflammation in blood values might be comparatively low [24]. Accordingly, this appears to be similar in the group of patients with acute leukemia in the current study. Furthermore, the initial therapy outcome, reflected by the extent and duration of pancytopenia and fever, was examined regarding its associations to the oral parameters. Although two types of significance were detected, the vast majority of examined parameters were found to be unaffected by treatment need and periodontal inflammation. Only one comparable examination is available, showing that poor oral health did not affect the induction outcome in children with acute leukemia [12]. This underlines the assumption that oral condition aside from dental infectious foci might be just a minor influential factor for hematological therapy outcomes. However, considering the literature [17], the patients within the current study were found to suffer from a comparably small PISA. Therefore, this small inflammatory burden might blur an effect on systemic factors and therapy outcomes.

A parameter of high clinical importance and relevance is the occurrence of infectious complications during induction therapy. A decision analysis model concluded that 318 out of every 1000 patients suffer from an infectious complication, of which 68 infections would be caused by oral source [10]. The occurrence of bacteraemia caused by oral diseases in leukemic patients undergoing chemotherapy is controversially discussed [11, 23]. On the one hand, periodontal diseases were found to be associated with an increased risk of infectious complications [21]. On the other hand, bacteraemia was not found to be associated with oral health in another study [11]. The current study was not able to find any evidence for associations between treatment need as well as periodontal inflammation and positive bacterial findings in blood or urine. Thereby, a real causative relationship appears difficult to assess.

The probably most important aspect seems to be the influence of oral health on the mortality of the patients. The above-mentioned analysis model assumes the possibility of additionally avoiding the death of 18 out of every 10000 patients by dental rehabilitation prior to chemotherapy [10]. This supports the recommendation of a dental clearance before therapy from the position paper of the joint task force of the Multinational Association of Supportive Care in Cancer/International Society of Oral Oncology (MASCC/ISOO) and the European Society for Blood and Marrow Transplantation (EBMT) [5]. However, an increased risk during dental intervention caused by the immunosuppression and risk for bleeding must be considered before induction therapy as well [6]. The current pilot study was not able to detect a relationship between remaining treatment need and periodontal inflammation after dental clearance with mortality. This underlines the occurrence of fatal complications due to oral status being a very rare condition, which might only be detectable in a very large cohort. Thereby, a causative relationship is difficult to assess. Although the dental and periodontal treatment need after dental clearance were not associated with therapy outcome, its prevalence was high. Therefore, an individualized, patient oriented dental care concept under consideration of the immune status and blood coagulation of patients seems to be the most likely recommendable strategy [6]. Thereby, the implication of prevention-oriented concept after chemotherapy with the aim of preserving teeth, if possible, could be a promising and safe approach [25]. Within this patient-oriented concept, the five core issues mentioned in the positions paper including prevention of infections, pain control, maintaining oral health, managing oral complications of treatment, and quality of life need to be addressed before, during, and after therapy [5].

Regardless of the missing association between oral conditions and outcome of induction therapy, dental rehabilitation and prevention remain a topic of high practical relevance. Early dental treatment and preventive special care should be applied within an interdisciplinary model. The integration of specialized dentists within oncologic centers might be a promising and helpful approach for the future to improve the dental care situation of patients with acute leukemia.

### 4.3. Strengths and Limitations

The current clinical study provides a comprehensive assessment of potential associations between dental/periodontal treatment need as well as periodontal inflammation with the initial treatment outcome and occurrence of complications of patients with acute leukemia, which previously underwent a dental clearance. Such data are limited in the literature but of high clinical importance. Nevertheless, there are several limitations to the current study. First, the small sample size is a major limitation. Especially in the subgroups, very small numbers of patients must be mentioned. Thus, the results of this pilot study must be seen as preliminary. Furthermore, design as a retrospective study limits the significance. However, the recruitment of patients with such severe disease is difficult due to their general disease burden. Thereby, based on the low prevalence of oral health related mortality mentioned in the literature [10] a very large cohort and a prospective design would be necessary to draw robust conclusions. Although a previous dental clearance was defined as an inclusion criterion, the content of the dental rehabilitation performed by the patient´s dentist and the presence of remaining foci, e.g., by radiographs, was not checked. Accordingly, sufficient dental clearance can only be assumed but cannot be guaranteed. In general, the definition of treatment need as presence of carious lesions or probing depth ≥3.5 mm is very strict and might include patients with a very small risk of infections. Moreover, the cut-off for the analysis of PISA needs to be mentioned as a limitation. While the analysis was performed by median to ensure subgroups with a balanced sample size, a more clinical oriented categorization depending on the severity of periodontal diseases might provide other results. Thereby, the new classification of periodontal diseases, with a staging and grading matrix and the differentiation between localized and generalized or stable and unstable periodontal conditions, might be of interest, respectively [26]. However, this would also lead to a very small sample size in the subgroups.

## 5. Conclusions

Within the limitations of this pilot study, dental and periodontal treatment need, as well as periodontal inflammation, is not associated with the outcome of induction therapy in adult patients with acute leukemia. Accordingly, the rehabilitation of dental foci prior to chemotherapy appears sufficient to avoid systemic complications. Because of the high prevalence of remaining treatment need after dental clearance, an individualized and prevention-oriented dental treatment concept should be applied after chemotherapy.

## Figures and Tables

**Table 1:** Patients' characteristics including age, gender, and treatment need, as well as PISA.

		Leukemia (*n* = 39)
Gender (*n* = 39)	Male *n* (%)	19 (49)
	Female *n* (%)	20 (51)

Age in years (*n* = 39) mv ± sd (median)		55.61 ± 17.01 (57)

Form of leukemia	Acute myeloid leukemia (AML)	26 (67)
	Acute lymphoblastic leukemia (ALL)	13 (33)

Dental treatment need (*n* = 39)	Yes	29 (75)
	No	10 (25)

Periodontal treatment need (*n* = 34)	Yes	25 (74)
	No	9 (26)

PISA (*n* = 34) mv ± sd (median)		153.18 ± 158.09 (98)

mv: mean value, sd: standard deviation, PISA: periodontal inflamed surface area.

**Table 2:** Initial blood parameters and therapeutic process depending on dental as well as periodontal treatment need.

Parameter		Periodontal treatment need			Dental treatment need		
		Yes (*n* = 25)	No (*n* = 9)	*p* value	Yes (*n* = 29)	No (*n* = 10)	*p* value
Initial blood parameters							
CRP (mg/l)		41.9 ± 41.7	21.1 ± 17.6	0.48	40.3 ± 39.7	46.8 ± 90.7	0.21
LDH (U/I)		642.6 ± 679.9	795.8 ± 1116.9	0.73	723.2 ± 870.4	597.8 ± 438.5	0.71
aPTT (sec)		28.4 ± 6.5	28.6 ± 2.7	0.90	29.1 ± 6.8	28.0 ± 3.6	0.58
Hemoglobin (g/dl)		9.8 ± 1.3	10.7 ± 2.3	0.29	10.1 ± 1.6	10.0 ± 1.6	0.95
Hematocrit (%)		28.6 ± 4.3	31.3 ± 6.8	0.29	29.6 ± 4.9	29.1 ± 5.3	0.78
Initial therapy parameters							

Erythrocytes (10³/*μ*l)	Initial	3.2 ± 0.6	3.5 ± 0.9	0.51	3.4 ± 0.7	3.2 ± 0.6	0.31
	3 days	3.4 ± 1.2	2.5 ± 1.7	0.85	3.1 ± 0.9	4.0 ± 1.9	0.14
	7 days	2.9 ± 0.5	2.4 ± 1.6	0.21	3.1 ± 1.0	2.9 ± 0.7	0.33

Thrombocytes (10³/*μ*l)	Initial	66.4 ± 69.1	153.4 ± 136.9	0.07	67.7 ± 76.0	145.4 ± 133.6	0.12
	3 days	59.5 ± 52.8	147.4 ± 135.4	0.19	68.0 ± 77.7	118.3 ± 110.2	0.63
	7 days	43.0 ± 44.5	131.3 ± 108.5	**0.03**	53.2 ± 67.1	83.3 ± 83.6	0.50

Leukocytes (10³/*μ*l)	Initial	18.4 ± 35.7	35.3 ± 59.7	0.31	25.2 ± 46.3	15.3 ± 24.1	0.50
	3 days	14.4 ± 27.6	31.1 ± 65.5	0.77	19.9 ± 41.2	19.2 ± 41.0	0.89
	7 days	6.9 ± 15.3	10.8 ± 12.1	0.07	8.3 ± 15.6	2.7 ± 3.2	0.82

CRP (mg/l) max		155.0 ± 95.1	102.4 ± 97.4	0.23	149.7 ± 88.4	198.2 ± 145.0	0.39

Duration of erythropenia in days		38.6 ± 22.6	28.7 ± 22.4	0.33	38.0 ± 21.4	40.0 ± 31.5	0.83

Duration of thrombopenia in days		31.7 ± 19.4	23.1 ± 16.9	0.28	29.6 ± 17.7	29.3 ± 23.0	0.97

Duration of leukopenia in days		26.8 ± 18.9	15.1 ± 11.1	0.09	23.5 ± 17.4	25.6 ± 18.2	0.59

Duration of neutropenia in days		15.9 ± 16.7	6.4 ± 7.9	0.11	13.6 ± 15.4	16.9 ± 15.3	0.49

Duration of fever in days		13.0 ± 13.0	9.3 ± 9.7	0.45	9.1 ± 7.8	25.8 ± 22.5	0.06

Positive microbiological findings	Blood	12/20 (60%)	2/5 (40%)	0.62	13/23 (57%)	5/6 (83%)	0.36
	Urine	13/21 (62%)	0/3 (0%)	0.08	9/20 (45%)	6/7 (86%)	0.09

CRP: C-reactive protein, LDH: lactate dehydrogenase, aPTT: activated Partial Thromboplastin Time, significant results are highlighted in bold (*p* < 0.05).

**Table 3:** Initial blood parameters and therapeutic process depending on PISA.

Parameter		PISA		
		>98 mm^2^ (*n* = 17)	≤98 mm^2^ (*n* = 17)	*p* value
Initial blood parameters				
CRP (mg/l)		35.4 ± 40.8	38.9 ± 36.6	0.53
LDH (U/I)		610.3 ± 789.3	760.9 ± 817.6	0.18
aPTT (sec)		29.5 ± 2.4	27.6 ± 7.5	0.13
Hemoglobin (g/dl)		9.5 ± 1.3	10.5 ± 2.0	0.06
Hematocrit (%)		27.8 ± 3.6	30.8 ± 6.0	0.09
Initial therapy parameters				

Erythrocytes (10³/*μ*l)	Initial	3.1 ± 0.5	3.5 ± 0.8	0.13
	3 days	3.0 ± 0.5	3.9 ± 1.5	**0.01**
	7 days	2.9 ± 0.4	3.4 ± 1.0	0.25
Thrombocytes (10³/*μ*l)	Initial	73.4 ± 76.3	104.8 ± 115.1	0.72
	3 days	61.1 ± 50.6	101.6 ± 111.7	0.56
	7 days	42.0 ± 34.7	79.3 ± 90.5	0.31
Leukocytes (10³/*μ*l)	Initial	13.2 ± 28.7	32.5 ± 52.9	0.34
	3 days	14.0 ± 31.0	22.6 ± 46.2	0.72
	7 days	8.5 ± 18.6	6.9 ± 9.7	0.31

CRP (mg/l) max		135.2 ± 101.6	153.0 ± 93.2	0.61

Duration of erythropenia in days		30.6 ± 19.7	43.1 ± 24.5	0.09

Duration of thrombopenia in days		28.6 ± 19.2	31.2 ± 19.3	0.70

Duration of leukopenia in days		26.6 ± 20.7	21.6 ± 14.6	0.65

Duration of neutropenia in days		16.3 ± 18.3	11.1 ± 11.8	0.47

Duration of fever in days		8.9 ± 8.1	15.4 ± 15.1	0.17

Positive microbiological findings	Blood	7/14 (50%)	7/11 (64%)	0.69
	Urine	7/14 (50%)	6/10 (60%)	0.70

CRP: C-reactive protein, LDH: lactate dehydrogenase, aPTT: activated Partial Thromboplastin Time, significant results are highlighted in bold (*p* < 0.05).

**Table 4:** Therapy associated complaints and mortality depending on treatment need and PISA.

Parameter		Weakness	Tachycardia	Vertigo/nausea	Dyspnea	Deceased		Reason for death		
						Yes	No	Cancer	Infection	Others
Dental treatment need	Yes	14/19 (48%)	9/29 (31%)	12/29 (41%)	7/29 (24%)	5/29 (17%)	24/29 (83%)	2	2	1
	No	3/8 (38%)	1/8 (13%)	4/8 (50%)	1/8 (13%)	1/10 (10%)	9/10 (90%)	1	0	0
	*p* value	0.70	0.40	0.71	0.66	0.99		—		

Periodontal treatment need	Yes	13/25 (52%)	6/25 (24%)	11/25 (44%)	6/25 (24%)	5/25 (20%)	20/25 (80%)	3	1	1
	No	3/8 (38%)	3/8 (38%)	2/8 (25%)	1/8 (13%)	1/9 (11%)	8/9 (89%)	0	1	0
	*p* value	0.69	0.65	0.43	0.65	0.99		—		

PISA	>98 mm^2^	7/17 (41%)	4/17 (24%)	7/17 (41%)	5/17 (29%)	4/17 (24%)	13/17 (76%)	2	1	1
	≤98 mm^2^	9/16 (56%)	5/16 (31%)	6/16 (38%)	2/16 (13%)	2/17 (12%)	15/17 (88%)	1	1	0
	*p* value	0.49	0.71	0.99	0.40	0.66		—		

PISA: periodontal inflamed surface area.

## Data Availability

No data were used to support this study.
